# Convergent acoustic community structure in South Asian dry and wet grassland birds

**DOI:** 10.1242/bio.058612

**Published:** 2021-06-22

**Authors:** Sutirtha Lahiri, Nafisa A. Pathaw, Anand Krishnan

**Affiliations:** Department of Biology, Indian Institute of Science Education and Research (IISER) Pune, Pashan Road, Pune 411008, India

**Keywords:** Grasslands, India, Acoustic community, Community bioacoustics, Signal space, Birds

## Abstract

Although the study of bird acoustic communities has great potential in long-term monitoring and conservation, their assembly and dynamics remain poorly understood. Grassland habitats in South Asia comprise distinct biomes with unique avifauna, presenting an opportunity to address how community-level patterns in acoustic signal space arise. Similarity in signal space of different grassland bird assemblages may result from phylogenetic similarity, or because different bird groups partition the acoustic resource, resulting in convergent distributions in signal space. Here, we quantify the composition, signal space and phylogenetic diversity of bird acoustic communities from dry semiarid grasslands of northwest India and wet floodplain grasslands of northeast India, two major South Asian grassland biomes. We find that acoustic communities occupying these distinct biomes exhibit convergent, overdispersed distributions in signal space. However, dry grasslands exhibit higher phylogenetic diversity, and the two communities are not phylogenetically similar. The Sylvioidea encompasses half the species in the wet grassland acoustic community, with an expanded signal space compared to the dry grasslands. We therefore hypothesize that different clades colonizing grasslands partition the acoustic resource, resulting in convergent community structure across biomes. Many of these birds are threatened, and acoustic monitoring will support conservation measures in these imperiled, poorly-studied habitats.

This article has an associated First Person interview with the first author of the paper.

## Abstract (precis)

**Summary:** Acoustic communities of different grassland biomes exhibit convergent signal space in spite of dissimilar species compositions.

## INTRODUCTION

The acoustic signals of different bird species may diverge to minimize competitive overlap, leading to overdispersion or uniform distribution of species within acoustic signal space. Each species within the acoustic community occupies a different region of this space (Krishnan, 2019; Luther, 2009; Nelson and Marler, 1990). However, the role of community phylogenetic structure in driving these signal space patterns remains poorly understood. To illustrate, if biogeographically distinct acoustic communities each partition the acoustic resource, they are predicted to exhibit convergent distributions in trait space, in spite of dissimilar phylogenetic compositions. Alternatively, communities may possess similar or divergent distributions in trait space simply as a consequence of phylogenetic similarity or dissimilarity (Cavender-Bares et al., 2004; Webb, 2000; Webb et al., 2002). Quantifying the species compositions of different bird acoustic communities, their respective signal (or trait) spaces, and phylogenetic similarity are necessary to test these predictions.

Grassland habitats are widespread across both tropical and temperate regions of the world, yet are highly threatened by habitat destruction (Nerlekar and Veldman, 2020). The loss of habitat is driving steep declines in grassland bird populations, many of which have highly specific habitat requirements. Thus, bird communities serve as a powerful indicator of ecosystem health (Correll et al., 2019; Dutta et al., 2011; Hill et al., 2014; Rahmani, 2012; Vickery and Herkert, 1999). However, until recently, grassland birds, particularly in tropical regions, have received relatively little study (Krishnan, 2019). This lacuna is pronounced in the Indian subcontinent, which possesses diverse grassland habitats (Ratnam et al., 2016) occurring along a range of rainfall regimes. The most widespread grassland biomes in this region include semiarid dry grasslands such as those in northwest India, and wet alluvial floodplain grasslands of the Gangetic-Brahmaputra floodplains (Dabadghao and Shankarnarayan, 1973). Bird species are thought to have speciated across the boundary between dry and wet grasslands (Ripley and Beehler, 1990), but there have been no detailed studies of their acoustic communities. An examination of community composition and phylogenetic similarity across these two biomes presents an opportunity to understand general principles underlying the assembly of acoustic communities, and also to establish a baseline for non-invasive monitoring.

**Fig. 1.**
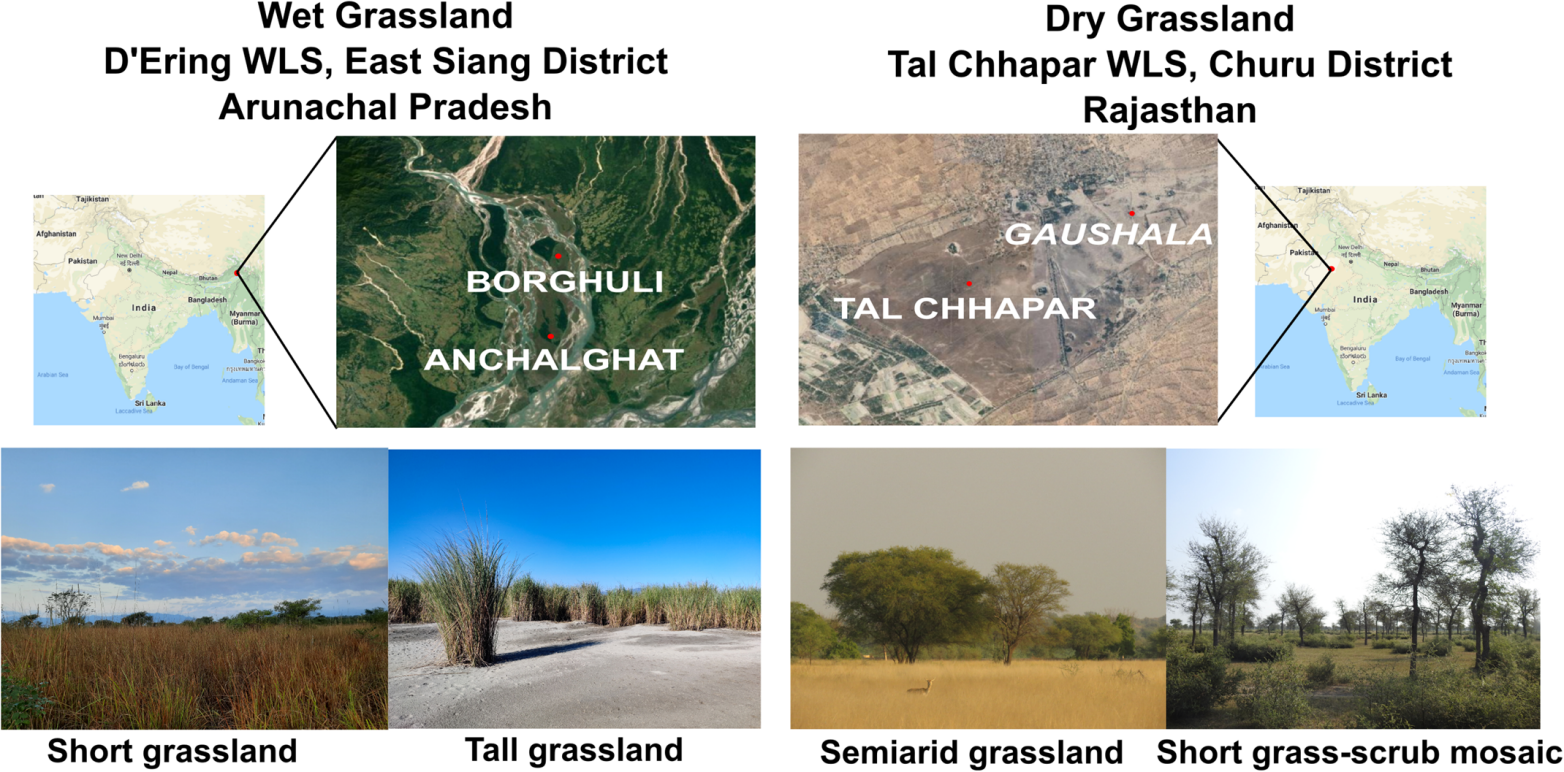
**Locations of the two sanctuaries, showing the various habitats sampled.** Photos courtesy of Taksh Sangwan (left) and Ram Mohan (right).

Here, we study the avian acoustic communities of dry grasslands in northwest India, and of wet floodplain grasslands in northeast India (Fig. 1). First, we assess the species compositions and diversity of birds in these acoustic communities. Secondly, we quantify the signal space occupied by vocal birds in each habitat, and assess whether they exhibit similar distributions in signal space. Finally, we test whether community structure arises from phylogenetic similarity between the communities (similar species or close relatives in each habitat), or from different bird groups expanding to fill the same signal space (i.e. phylogenetically dissimilar communities). Our findings, some of the first detailed acoustic data from these habitats, have great value in long-term conservation monitoring of threatened grassland biomes and their unique, diverse avifauna.

## RESULTS

### Avian acoustic communities of wet and dry grasslands

**Fig. 2.**
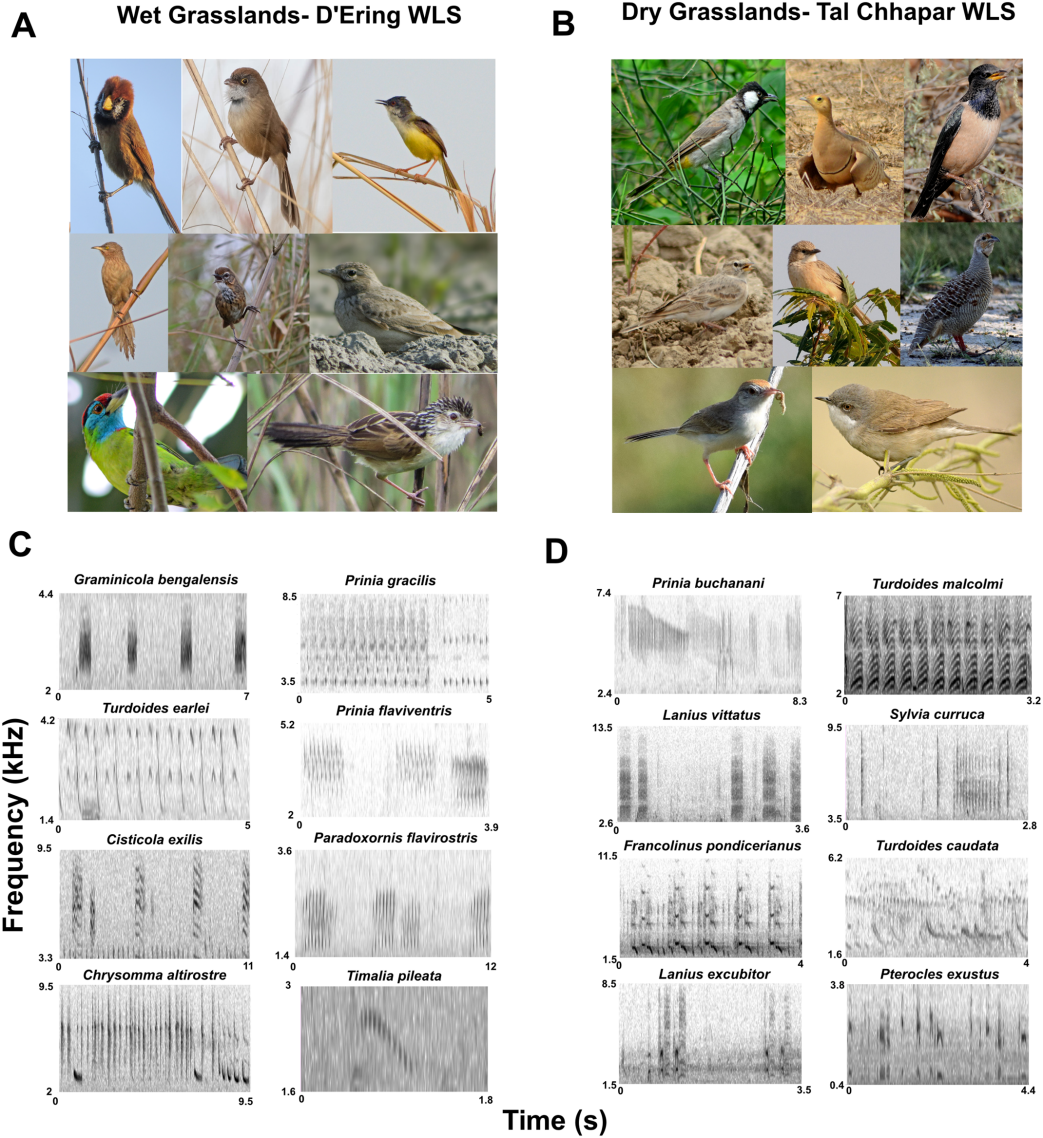
**(A,B) Some of the birds comprising the acoustic community in wet (A) and dry (B) grasslands.** (A) Top row, left to right, photographer's name in brackets: *Paradoxornis flavirostris* (Taksh Sangwan), *Chrysomma altirostre* (Roon Bhuyan), *Prinia flaviventris* (S.L.), middle row: *Turdoides earlei* (S.L.), *Pellorneum palustre* (Siva R.), *Alaudala raytal* (S.L.), bottom row: *Psilopogon asiaticus* (S.L.), *Graminicola bengalensis* (Taksh Sangwan). (B) Top row: *Pycnonotus leucotis* (S.L.), *Pterocles exustus* (S.L.), *Pastor roseus* (A.K.), middle row: *Calandrella brachydactyla* (S.L.), *Turdoides caudata* (A.K.), *Francolinus pondicerianus* (A.K.), bottom row: *Prinia buchanani* (A.K.), *Sylvia curruca* (S.L.). (C,D) Spectrograms of sample vocalizations recorded during data collection in wet (C) and dry (D) grasslands, with species names above each.

**Fig. 3.**
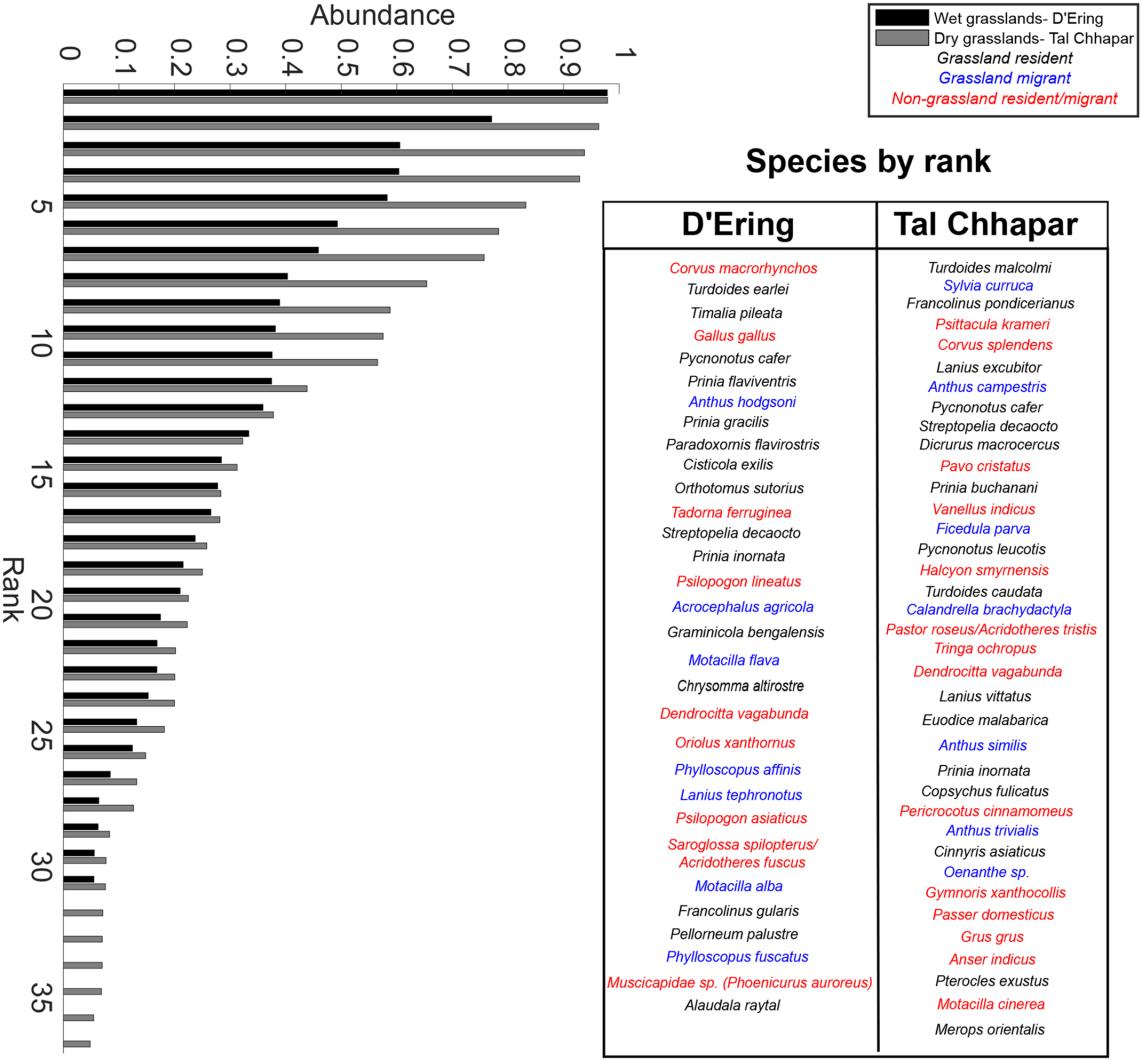
**Ranked abundance distributions for both acoustic communities using the acoustic abundance index.** The box contains species names in order of abundance, and color-coded by habitat preference.

We identified vocalizations of 52 bird species in wet grasslands, and 68 in dry grasslands (Supplementary Material, Fig. S1). Of these, 31 and 37 species (32 and 38 if both starling species are considered separately, see Materials and Methods) were recorded in >5% of 10-min samples, and we considered these to constitute the acoustic community (see Fig. 2 for examples). These species, classified according to habitat, are listed in Fig. 3. Jaccard's similarity between the dry and wet grassland acoustic communities was 0.06, as they only shared four species (*Pycnonotus cafer*, *Prinia inornata*, *Streptopelia decaocto* and *Dendrocitta vagabunda*), thus indicating large differences in community species composition.

Ranked-abundance distributions (Fig. 3) demonstrated that higher-ranked species in dry-grassland habitats possessed acoustic abundance indices above 0.75, whereas only two species in wet grasslands had abundance indices above 0.6. Evenness metrics E_Q_ and E_var_ were slightly higher for wet grasslands (after normalizing for overall species diversity). This suggests that the higher abundance of a few vocally common species in the dry grasslands resulted in slightly lower evenness across the acoustic community (E_Q_: wet grasslands 0.22, dry grasslands 0.19, E_var_: wet grasslands 0.66, dry grasslands 0.55).

### Wet and dry grasslands exhibit convergent signal space structure

**Fig. 4.**
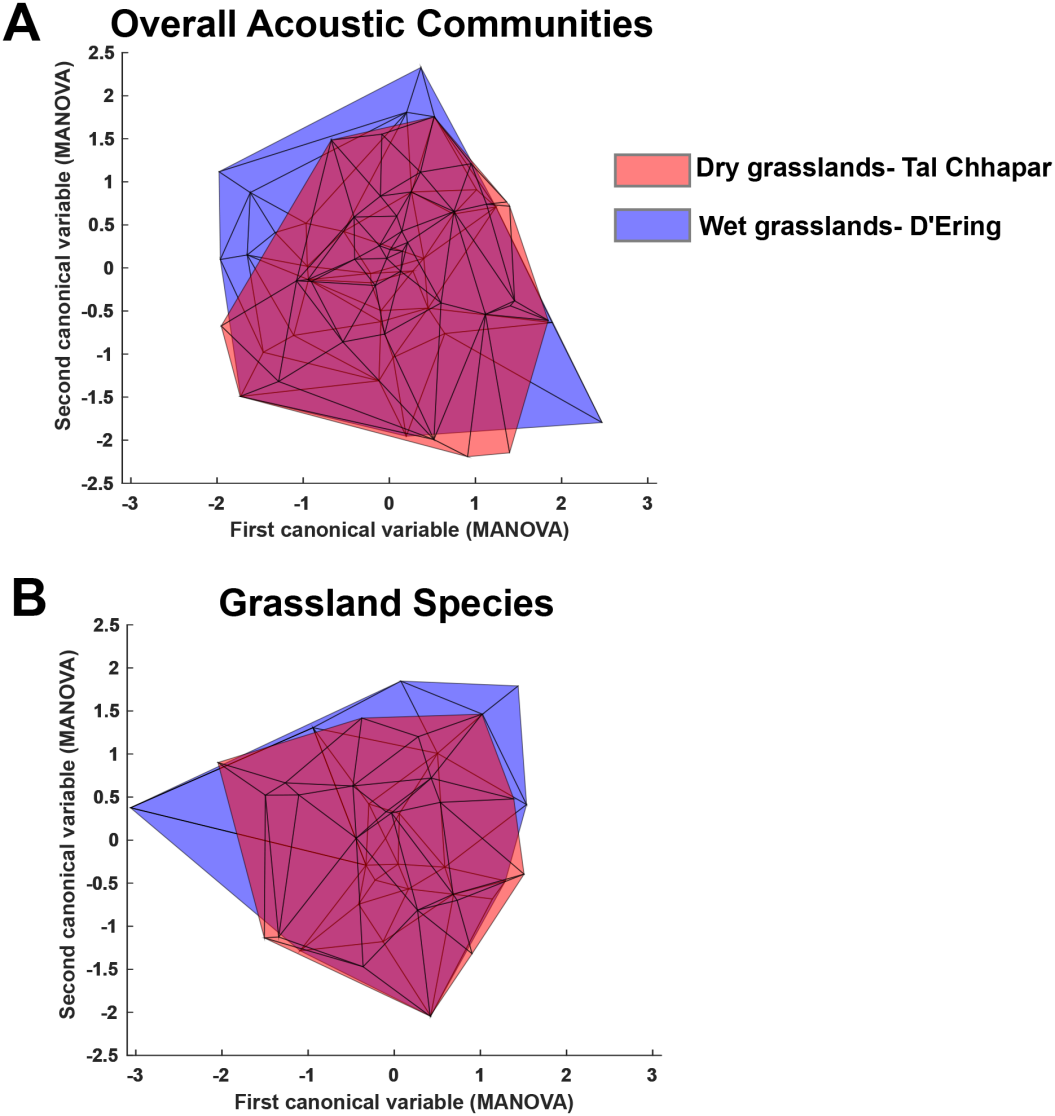
**Biplot of the first two canonical variables from a MANOVA on the principal components of acoustic parameters.** These parameters were measured for each species in the two acoustic communities to derive a signal space, plotted here as a minimum convex polygon. Note the overlap in polygons both when considering all species (A) and only grassland species (B).

Next, we compared the community signal space of wet and dry grassland acoustic communities. The first three principal components (PCs) of eight acoustic signal parameters accounted for about 85% of total variation (Supplementary Material), loading positively on frequency parameters (PC1), average entropy and bandwidth (PC2), and relative time of peak (PC3). Both communities exhibited a dispersed community structure, although the wet grassland community fit 100 randomized uniform distributions slightly better than the dry grassland community (Kolmogorov–Smirnov tests against 100 randomized uniform distributions: wet grasslands, all species=99%, grassland species=94%, dry grasslands, all species=72%, grassland species=64%; percentages indicate number of times out of a 100 where the observed distribution was similar to a uniform distribution at P greater than 0.05) (Supplementary Material) (Krishnan, 2019). This dispersion is consistent with divergent signals and low interspecific overlap. Furthermore, MANOVA on both the total acoustic community (Fig. 4A) and the grassland species alone (Fig. 4B) showed that the two acoustic communities occupied the same regions of signal space (all species: d.f. within groups=68, d.f. between groups=1, total d.f.=69, Wilk's lambda on PCs=0.967, *P*=0.53, on song parameters: lambda=0.82, *P*=0.13; grassland species: d.f. within groups=43, d.f. between groups=1, total d.f.=44, Wilk's lambda on PCs=0.99, *P*=0.93, on song parameters: lambda=0.81, *P*=0.41). This suggests that the slightly higher clustering observed in the dry grassland community is because of slightly higher species diversity within the same region of signal space. Both communities accumulate species at broadly similar rates with distance from the origin of PC space, further supporting this assertion (Fig. S2).

The findings above suggest that the two grassland biomes exhibit similar overdispersed distributions of species in acoustic signal space. Consistent with this, a comparison of the observed between-community nearest-neighbor distances (NNDs) to those obtained from 10,000 randomized null communities showed that the NNDs between dry and wet grassland acoustic communities were significantly lower than expected by chance (Z=−3.43, *P*<0.01). This result also held true when only considering grassland species (Z=−3.9, *P*<0.01), suggesting that both these grassland biomes, in spite of possessing very few species in common, exhibit similar dispersed distributions in acoustic signal space.

### Dry grassland acoustic communities exhibit higher phylogenetic diversity

**Fig. 5.**
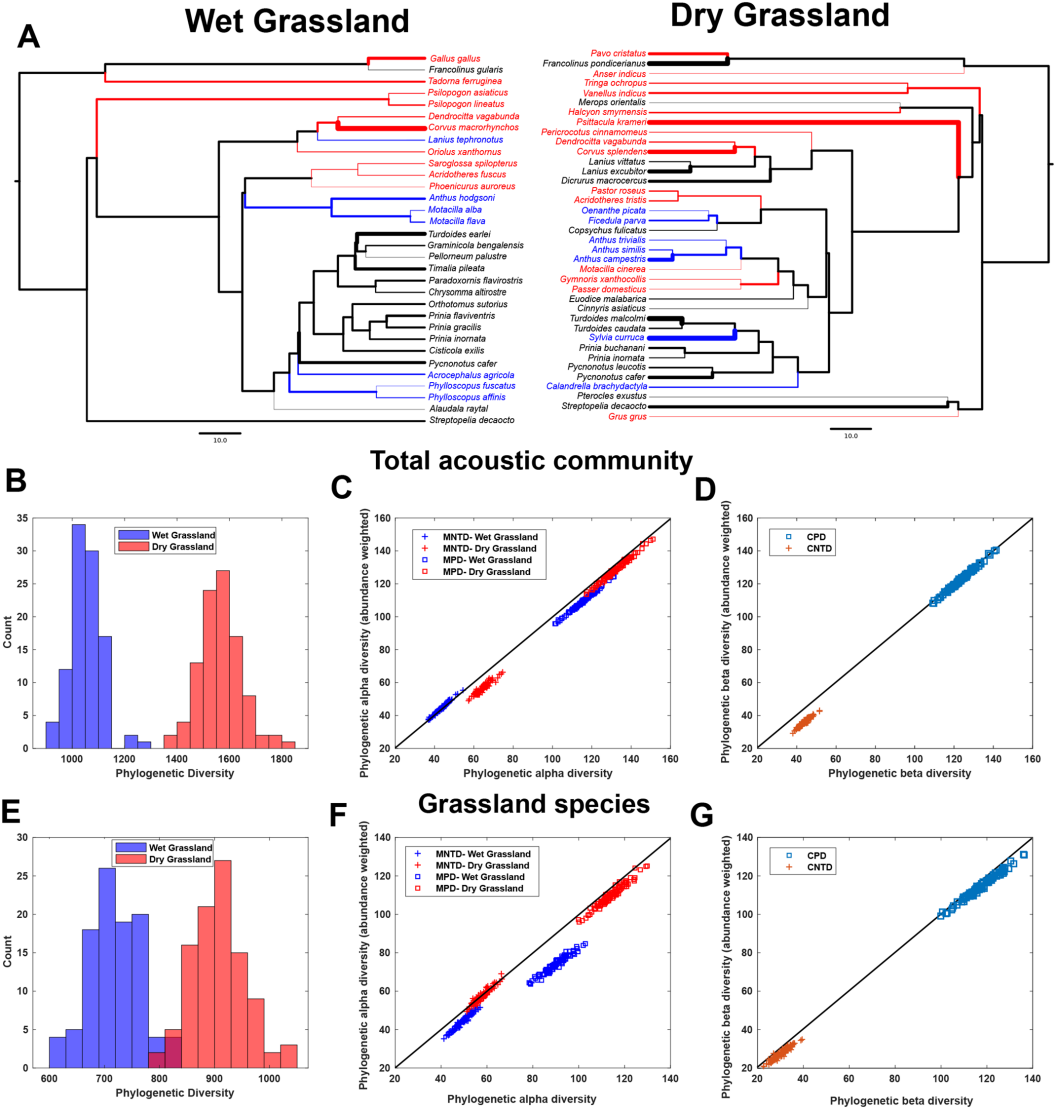
**(A) Majority-rule consensus trees representing the phylogenetic relationships of species in both acoustic communities, color-coded by habitat preference as in earlier figures.** Thickness of terminal branches is in proportion to abundance index for each species. (B–G) Phylogenetic alpha diversity (B,C,E,F) and beta diversity (D,G) metrics for both acoustic communities, both for all species (B–D) and grassland species (E–G). In C,D,F and G, indices are also calculated after weighting for abundance. In all scenarios, dry grasslands possess higher phylogenetic diversity.

Summarizing our results so far, the two grassland acoustic communities exhibit different species compositions, but convergent signal space structure. We next calculated measures of phylogenetic diversity (Fig. 5), to understand whether similarity in signal space was due to phylogenetic similarity of the acoustic communities. Three measures of alpha diversity, Faith's phylogenetic diversity, mean pairwise distance and mean nearest-taxon distance (PD, MPD, and MNTD), were all significantly higher for dry grasslands than wet grasslands (Wilcoxon signed-rank test, *N*=100; W=0, *P*<0.001 for all three), even after weighting for abundance (Fig. 5B,C). The same pattern held when considering only grassland species (Wilcoxon signed-rank test, *N*=100; W=0, *P*<0.001 for all three) (Fig. 5E,F). Phylogenetic beta-diversity (community pairwise distance and community nearest-taxon distance, CPD and CNTD) metrics were of roughly the same magnitude (or slightly lower, see Supplementary Material) as the corresponding alpha diversity metrics (Fig. 5D,G). Weighting for abundance significantly lowered both CPD and CNTD (Wilcoxon signed-rank test of raw versus weighted values, *N*=100; all species: CPD: W=4973, CNTD: W=5050, *P*<0.001, grassland species: CPD: W=5050, CNTD: W=5050, *P*<0.001). Overall, though, between-community phylogenetic distances remained broadly similar to within-community distances. A phylogenetic community dissimilarity (PCD) value of 1.71 (all species) and 1.69 (grassland species) indicated greater dissimilarity between the communities than expected by chance. The PCDc value (1.87 in both cases) suggested greater differences in species composition, and PCDp (the phylogenetic component) was 0.92 (all species) and 0.91 (grassland species) (Supplementary Material). Thus, the acoustic communities did not exhibit significant phylogenetic similarity (Ives and Helmus, 2010).

**Fig. 6.**
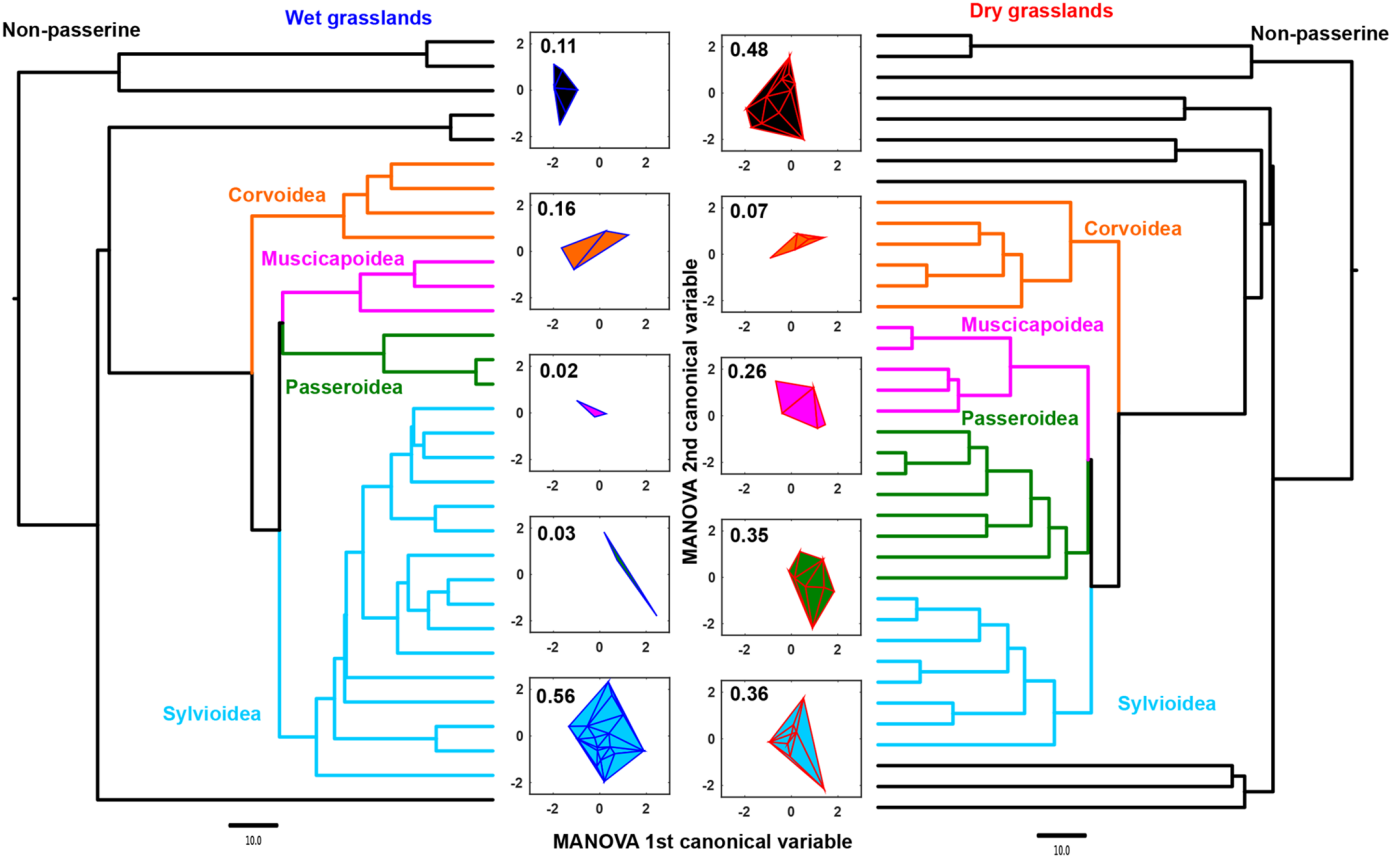
**Consensus trees for each acoustic community with the major clades color-coded, and their respective signal spaces represented by minimum convex polygons in the center.** Wet grasslands are on the left (blue outline on polygons), dry grasslands on the right (red outline), and the polygons are colored according to the clade they represent. The numbers above each polygon represent its area divided by the area of the minimum convex polygon containing all species (see Fig. 4). Note the dominance of the Sylvioidea in wet grasslands.

Higher phylogenetic diversity in dry grasslands, together with a lack of phylogenetic similarity suggests that phylogenetic considerations alone do not explain convergent community structure. Because these acoustic communities are largely composed of passerine birds, we considered all non-passerines as one group, and the major passerine clades represented in our dataset (Corvoidea, Muscicapoidea, Passeroidea and Sylvioidea) as the other four groups. The dry grassland acoustic community contained 11, 6, 5, 8 and 8 species from each of these five groups, respectively. However, the wet grassland acoustic community contained 6, 4, 3, 3, and 16 species, respectively (considering the two starling species separately in each). The Sylvioidea (babblers, warblers, bulbuls and larks) (Alström et al., 2006) accounted for half the species in the wet grassland acoustic community, with a concomitant expansion in signal space compared to their dry grassland counterparts (Fig. 6). This expansion of the Sylvioidea in wet grasslands appears to drive convergent signal space and community structure, in spite of the lower phylogenetic diversity in wet grasslands.

## DISCUSSION

To summarize, we recorded diverse acoustic communities from both wet and dry grasslands in India. Although the two communities were not significantly phylogenetically similar, both exhibited convergent distributions in acoustic signal space. The Sylvioidea appear to have expanded in wet grassland signal space to occupy the same acoustic resource as the dry grassland community, even though overall phylogenetic diversity is lower in wet grasslands. In spite of lower phylogenetic diversity, signal space of wet grasslands is convergent with dry grasslands, both communities exhibiting overdispersion in signal space. Inter-community NNDs are lower than expected by chance, which is indicative of each species having a counterpart in the signal space of the other community. This may occur because closely related species replace each other across habitats. Their signals may resemble each other owing to their shared ancestry, thus leading to convergence in signal space due to phylogenetic similarity (Cardoso and Price, 2010; Tobias et al., 2014).

However, our phylogenetic analyses suggest the converse. Firstly, dry grassland acoustic communities have higher phylogenetic diversity, and second, the two grassland communities are no more or less phylogenetically similar than expected by chance (PCDp close to 1). The results of PCD hold even when considering all recorded species (and not just those recorded often enough to form the acoustic community, PCDp=0.9, see Supplementary Material), so our results are robust to the definition of the acoustic community that we employ here. This suggests that although some close relatives may replace each other between habitats (further supported by the values of between-community CPD and CNTD being within the same range as the within-community MPD and MNTD), convergence is also responsible for the observed similarity in signal space. Some species are replaced by congeners between dry and wet grasslands (e.g. *Francolinus*, *Turdoides*) (Ali and Ripley, 1997; Rasmussen and Anderton, 2005; Ripley and Beehler, 1990). However, others are unique to each type of grassland. For example, *Pterocles* is found only in semiarid habitats, and *Paradoxornis* only in wet grasslands (Ali and Ripley, 1997; Grimmett et al., 1998; Rahmani, 2012; Rasmussen and Anderton, 2005). The fact that distributions of species in signal space are similar in spite of these phylogenetic differences is consistent with convergence in signal space. A similar pattern holds regardless of whether we consider strictly grassland species, or the entire acoustic community, indicating that the presence of villages or wooded patches does not change patterns in signal space.

The Sylvioidea (babblers, warblers, bulbuls and larks in our dataset), passerine birds of roughly similar body size, comprises half of the wet grassland acoustic community. This overrepresentation compared to the dry grasslands is accompanied by an increase in the signal space occupied by this clade (Fig. 6), such that the overall signal space of the two communities is convergent. Because these species are largely grassland-dwelling, this pattern holds even when considering only grassland species. The eastern Himalayas and their foothills possess the highest babbler diversity in the Indian subcontinent (Srinivasan et al., 2014). This biogeography may partly explain why the acoustic community of the wet grasslands (occupying this region) is dominated by this clade. The expansion of the Sylvioidea, resulting in convergent signal space with northwestern dry grasslands, is a compelling indicator of the presence of ‘niches’ in acoustic signal space. This is also supported by the fact that this one clade has expanded in signal space, in spite of being roughly similar in body size. Although we focus on community structure rather than signal partitioning, our analyses suggest that species within each community are dispersed across acoustic signal space, a pattern that is consistent with signal partitioning (Fig. 4). Thus, we hypothesize that grassland bird acoustic communities assemble by partitioning the available acoustic signal space (analogous to filling available niches) (Price et al., 2014), resulting in convergent community structure across different grassland habitats. This pattern may potentially arise owing to multiple drivers of signal evolution, including to minimize acoustic interference between relatives (Schmidt et al., 2013), as an indirect outcome of morphological divergence between coexisting species (Krishnan and Tamma, 2016), or due to similarities in habitat structure (Marten and Marler, 1977). In addition, temporal partitioning between vocal species (minimizing overlap by singing at different times) (Luther, 2008) may further accentuate the separation between species in signal space, and future studies will investigate temporal patterns of interspecific song overlap. The drivers of acoustic community assembly and structure are thus a compelling subject for future study. Quantifying community-level signal space is a valuable way to establish patterns using passive acoustic data. Thus, we propose combining these methods with phylogenetic analyses to study spatiotemporal change in global biodiversity.

Because of the multitude of threats facing tropical and subtropical grasslands in India (Ratnam et al., 2016), and the generally poor status of knowledge about their birds, bioacoustics studies have great potential to illuminate their natural history and conservation status (Blumstein et al., 2011; Campos-Cerqueira and Aide, 2016; Raynor et al., 2017; Sugai et al., 2019). This is particularly true of the wet grasslands of the Brahmaputra floodplains, which have suffered extensive conversion to agriculture (Jha et al., 2018; Rahmani, 2016b). Many threatened birds in these landscapes remain very poorly studied, and protected areas such as our two study sites represent strongholds for many grassland bird species. We recorded multiple globally threatened species (e.g. *Laticilla cinerascens*, *Paradoxornis flavirostris*, *Chrysomma altirostre*, *Pellorneum palustre*, *Francolinus gularis* and *Graminicola bengalensis*) (Rahmani, 2012) in our study, many of them being frequent vocal components of their respective acoustic communities. Our data on acoustic community structure shed valuable light on their habitat preferences, and the bird species they coexist with, using completely non-invasive tools. Community bioacoustics studies have great potential to inform conservation policy for threatened grassland habitats, as well as monitoring the long-term health of these fragile ecosystems.

## MATERIALS AND METHODS

### Study sites

We conducted fieldwork in protected areas containing some of the best-preserved semiarid and floodplain grasslands in India (which we refer to as ‘dry grassland’ and ‘wet grassland’ henceforth) (Fig. 1). For wet grasslands, we carried out acoustic sampling in the D'Ering Wildlife Sanctuary in East Siang district, Arunachal Pradesh (27°51′-28°5′ N, 95°22′-95°29′ E). This sanctuary consists of seasonally inundated riverine islands, or *chaporis*, with a *Phragmites*-*Saccharum*-*Imperata* type grassland structure (Dabadghao and Shankarnarayan, 1973; Rahmani, 2016a). Within this sanctuary, we sampled in the Borghuli and Anchalghat ranges, which contain a mixture of two grassland types: tall (3–4 m) grassland, and shorter (1 m) grassland. These grasslands are in close proximity to water, with human habitation on the other side of the Siang River.

For dry grasslands, we sampled in the Tal Chhapar Wildlife Sanctuary in Churu district, Rajasthan (27°47′ N, 74°26′ E). This sanctuary is located on the edge of the Thar Desert, containing xerophilous *Dichanthium*-*Cenchrus*-*Lasiurus* type grasslands, with intermixed scrub of species such as *Capparis* and *Prosopis* (Dabadghao and Shankarnarayan, 1973; Kaur et al., 2020) and human-made water bodies. The village of Chhapar exists at the border of this relatively small sanctuary. We sampled sites within the sanctuary, as well as grassland habitats in an adjacent community grazing area (or *gaushala*). This latter site possessed additional grassland habitat with interspersed shrubs and thorny bushes, and also harbored many of the same bird species as found in the sanctuary. Thus, although it lay outside the protected area, it may be considered an extension of the same grassland habitats found in the sanctuary itself.

### Fieldwork and acoustic sampling

We sampled winter acoustic communities in both grassland habitats, partly to include winter migrant birds (Grimmett et al., 1998; Rasmussen and Anderton, 2005) in our analysis of acoustic communities, but primarily because the grasslands at D'Ering WLS are inundated and inaccessible for much of the summer-monsoon season (Rahmani, 2016a). Winter migrants in the tropics are frequently vocal, and recent research suggests that they do not change the signal space of tropical bird acoustic communities (Krishnan, 2019). Therefore, the winter season is the best time to directly compare different grassland acoustic communities at a similar time of the year. Our study at Tal Chhapar was conducted in late November-early December 2019, and our study at D'Ering shortly afterward in early January 2020, within the winter migrant season (Krishnan, 2019). Prior to sampling, we conducted reconnaissance trips to these sites, and these enabled us to identify suitable recording sites, as well as familiarize ourselves with species vocalizations for subsequent identification in recordings.

Following published methodology described previously (Krishnan, 2019; Luther, 2009), we recorded the dawn singing activity in grassland habitats (see Fig. S1 for examples) and used this to quantify the composition, diversity and structure of acoustic communities. Bird song was recorded at a 44.1 kHz sampling rate using either a SongMeter SM4 (Wildlife Acoustics Inc., Maynard, MA, USA, omnidirectional with frequency sensitivity from 20Hz–48 kHz) or a Sennheiser (Wedemark, Germany) ME62 omnidirectional microphone (20Hz–20 kHz) connected to a Zoom (Tokyo, Japan) H6 recorder. In total, we sampled six distinct recording locations in D'Ering WLS, three in the Anchalghat range and three in the Borghuli range. At Tal Chhapar WLS, we sampled five sites, three in the main sanctuary and two in the *gaushala*. Sites were selected based on the availability of suitable vegetation (extensive patches of grassland/scrub, and a safe place for the recorders). At each site, we began recording at 06:00 (early dawn, approximately the sunrise time in both locations at this season), and recorded for between 2 and 3 h (depending on weather and the time taken to reach recording sites to take down the recorders). We took care to ensure that the presence of observers did not affect bird singing activity, placing the recorders away from human activity and returning only to take down the recorders after sampling. Because of the open nature of grassland habitats (where most birds are within a few meters of the ground), and our strategy of sampling multiple recording sites in each habitat, we were very unlikely to miss species vocalizations during sampling (Krishnan, 2019). Therefore, we consider these data to be a comprehensive sample of vocal activity at these sites.

In D'Ering WLS, we sampled each site twice (on separate days), resulting in a total of 36 h of audio over 12 individual recordings. At Tal Chhapar, because there were a smaller number of sampling locations where we could place recorders, we sampled two sites four times each, a third three times and obtained two recordings from the *gaushala*. This was a total of 13 individual recordings representing approximately 36 h of audio. In one of these recordings, an equipment malfunction left us with only 1 h of audio, and so we excluded it from further analyses. Therefore, we obtained a comparable amount of audio data from dry and wet grasslands, and of similar or larger sample sizes than published studies on bird acoustic communities (Krishnan, 2019; Luther, 2009; Robert et al., 2019). In addition, by recording at multiple sites across both protected areas, we were able to obtain comprehensive coverage of the acoustic community across these landscapes.

### Acoustic community analysis

After dividing each recording into 10-min segments, we identified all bird species vocalizing in each segment and constructed presence-absence matrices (one matrix for each individual recording, i.e. 12 each from D'Ering and Tal Chhapar), where a value of 1 meant the species’ vocalizations were detected (regardless of which call was emitted), and a 0 indicated absence. Because of the sedentary, territorial nature of most of these species, we consider this window appropriate for census purposes (Ali and Ripley, 1997). Two of us censused the data to minimize individual biases in detection, using the spectrogram windows and slow-motion play features in Raven Pro, and also cross-verified species identity with each other to minimize identification uncertainties. Using recordings, our observations during sampling and during our reconnaissance trips (see above), together with online song databases, we were able to identify most vocalizations down to species. The use of the 10-min sampling window was also helpful here, as this minimized the likelihood that chance noise events would confound identification. The few remaining unidentified vocalizations were almost all single detections (much less than 1% of the total) that do not, therefore, influence our community-level analysis. For further analysis, we considered species that had been detected in at least 5% of total 10-min samples (considering the first 2 h of all recordings, see below). Next, we categorized these species (based on published information about their habitats) into grassland residents, grassland migrants and non-grassland species (both resident and migrant) (Ali and Ripley, 1997; del Hoyo et al., 2014; Grimmett et al., 1998; Rasmussen and Anderton, 2005). The first two categories consisted of species that regularly use grassland habitats, even if not entirely confined to them. The third category contained birds traditionally considered forest-inhabiting, waders and waterfowl, and birds from nearby human habitations (Supplementary Material). Birds in this last category may still utilize grassland habitats occasionally (Rasmussen and Anderton, 2005), and their sounds may form an integral part of the acoustic community. Thus, to control for any subjectivity arising from this classification, we performed all analyses both on the subset of grassland species, as well as the entire acoustic community, and observed no differences in broad patterns.

For each species within the two acoustic communities, we determined an ‘acoustic abundance index’, using the first 2 h of each individual recording (roughly 24 h of audio total from each community and 48 h in total, 12 10-min samples per recording, and 12 recordings for each community), based on published methods (Krishnan, 2019). We used the first 2 h of each recording in this analysis to ensure a consistent sample size to calculate acoustic abundance; bird singing activity was typically higher in this time period. The acoustic abundance index represents the probability of detecting a species’ vocalizations if one 10-min sample were drawn at random from each of the 12 presence-absence matrices (one for each sample). We performed this random draw 10,000 times, and took an average of the proportion of samples that contained the species, following a published workflow (Krishnan, 2019). Next, we constructed ranked-abundance curves for each habitat (Avolio et al., 2019), and calculated Jaccard's similarity index (i.e. shared vocal species between dry and wet grasslands), and the evenness metrics E_Q_ and E_var_, (Smith and Wilson, 1996) using the codyn (Hallett et al., 2020) package in R (R Core Team, 2013). For both metrics, a value of 0 implies a non-even community and 1 a perfectly even community.

### Signal parameter space and acoustic community structure

In order to derive the signal space of wet and dry grasslands, we calculated note parameters for each species in the acoustic community using recordings from the databases Xeno-Canto (https://www.xeno-canto.org/) and AvoCet (https://avocet.integrativebiology.natsci.msu.edu/) (Supplementary Material). This was because we wanted to ensure a good signal:noise ratio for reliable estimates of note parameters, which was not always possible from passive recordings. We curated this dataset to only include recordings from the same sanctuaries or adjacent contiguous areas (which are popular birding areas) of grassland where possible, and also digitized multiple recordings per species to ensure more accurate representation in signal space. This also ensured that vocal parameters measured were from the same song notes we detected at our field sites. Because both these databases use different file types, it is likely that some variation in frequency parameters may be introduced owing to file compression (Araya-Salas et al., 2019), even though most of the files we digitized were sourced from Xeno-Canto (Supplementary Material). However, this variation is within the range for each species, and is thus unlikely to significantly affect interspecific comparisons and community patterns. For all species, and particularly migrants, we additionally verified that labeled note types or calls were the same as those we detected in our recordings, thus ensuring reliability of our signal space to any confounding variation in the databases. After labeling the notes (10–20 notes per recording per species, depending on the number of notes in the recordings; we took care to digitize all notes in a species’ call or song) in Raven Pro 1.5 (Cornell Laboratory of Ornithology, Ithaca, NY, USA), we calculated the following eight parameters: average, maximum and minimum peak frequency, center frequency, 90% bandwidth, average entropy, note duration, and relative time of peak (i.e. the point during the note at which the peak frequency occurred) (Supplementary Material). These parameters were chosen from published studies that quantified community signal space (Chitnis et al., 2020; Krishnan, 2019), which also calculated parameters at the level of notes in order to compare phylogenetically disparate species with very different call types. All measurements were made using a spectrogram window size of 512 samples, with an overlap size of 256 samples, and contrast and brightness settings at 50 (the default settings in Raven Pro). Taking a species average for each of these parameters, we performed a principal components analysis on the correlation matrix using MATLAB (Mathworks Inc., Natick, MA, USA), and used the principal component scores to test for patterns within community signal space (Luther, 2009). We performed three statistical analyses: first, we compared the principal component scores of dry and wet grassland acoustic communities to 100 randomly drawn uniform distributions spanning the same range of scores, using Kolmogorov–Smirnov tests (the kstest2 function in MATLAB) (Krishnan, 2019). By measuring the percentage of times each community fit to a uniform distribution (in this case, *P*<0.05 means they differed significantly from uniform), we tested whether they showed signatures of dispersion versus clustering in signal space, consistent with divergent acoustic signals within the community. Second, we tested whether the two communities occupied similar regions of signal space by testing whether their respective point clouds in principal component space were drawn from the same distribution, using a MANOVA with Wilk's lambda as the test statistic on the first three principal components (the manova1 function in MATLAB), and separately on the original parameters as well. The last test we performed built upon this previous one to also test whether the organization of points within principal component space (or signal space structure) was similar across the two acoustic communities. For this, we calculated the NND for species across communities (Krishnan, 2019; Luther, 2009). For each species in wet grassland, we calculated the Euclidean distance to its nearest neighbor in the dry grassland community. If the two communities exhibited similar patterns of community organization in signal space, then the average between-community NND should be lower than that of a randomly drawn community. Therefore, we constructed 10,000 randomly drawn ‘null communities’ (Chek et al., 2003) spanning the same range of principal component scores, calculated the average NND to each of these communities, and then calculated the Z score of the observed NND versus this distribution of null values (Krishnan, 2019). All three statistical tests were performed twice, once for the entire acoustic community and once for only the grassland species (see above).

### Phylogenetic diversity

After testing for patterns in community structure using the signal space of both acoustic communities, we tested whether both acoustic communities were phylogenetically similar. For this, we downloaded a meta-tree from the open-source Bird Tree of Life Project (https://birdtree.org/) (Jetz et al., 2012), containing 100 possible phylogenetic hypotheses for all the species within both acoustic communities. In the case of mixed-species starling flocks, we were unable to calculate separate abundance indices for each species because it was difficult to separate their vocalizations. Therefore, we included only one species from each habitat (*Acridotheres sp.*) in phylogenetic diversity analyses, although we included both in signal space calculations. When measuring phylogenetic diversity, we calculated values for each of the 100 possible trees, giving us a distribution of values (Krishnan, 2019). We then compared diversity metrics to each other using paired Wilcoxon signed-rank tests. Using commands within the picante (Kembel et al., 2010) package in R, we calculated three phylogenetic alpha-diversity (within-community) indices: Faith's PD, MPD and MNTD for each acoustic community, and two beta-diversity (between-community) indices: CPD, and CNTD (Tucker et al., 2017). In addition to the raw values, we also weighted phylogenetic diversity indices by the acoustic abundance index. Finally, to quantify whether the two acoustic communities exhibited congruent phylogenetic structure, we calculated the PCD between the two communities (Ives and Helmus, 2010), again using commands within the picante package. Using consensus trees for both the dry and wet grassland acoustic communities, we calculated PCD by comparison to 10,000 randomly reshuffled trees using the inbuilt permutation test in the pcd function of the picante package. This function outputs an overall PCD value, which is close to 1 if the two communities are as similar as expected by random chance, >1 if they are more dissimilar than expected, and <1 if they are more similar. The function additionally calculates the contribution of non-phylogenetic components (shared species, PCDc) and phylogenetic components (PCDp). This allowed us to assess whether the PCD value obtained arose because of shared species within the community versus similarities in phylogenetic structure. Again, we calculated all phylogenetic diversity metrics both for the entire acoustic community, and for grassland species.

## Supplementary Material

Supplementary information

## References

[BIO058612C1] Ali, S. and Ripley, S. D. (1997). *Handbook of the Birds of India and Pakistan*, 2nd ed. New Delhi: Oxford University Press.

[BIO058612C2] Alström, P., Ericson, P. G. P., Olsson, U. and Sundberg, P. (2006). Phylogeny and classification of the avian superfamily Sylvioidea. *Mol. Phylogenet. Evol.* 38, 381-397. 10.1016/j.ympev.2005.05.015 16054402

[BIO058612C3] Araya-Salas, M., Smith-Vidaurre, G. and Webster, M. (2019). Assessing the effect of sound file compression and background noise on measures of acoustic signal structure. *Bioacoustics* 28, 57-73. 10.1080/09524622.2017.1396498

[BIO058612C4] Avolio, M. L., Carroll, I. T., Collins, S. L., Houseman, G. R., Hallett, L. M., Isbell, F., Koerner, S. E., Komatsu, K. J., Smith, M. D. and Wilcox, K. R. (2019). A comprehensive approach to analyzing community dynamics using rank abundance curves. *Ecosphere* 10, e02881. 10.1002/ecs2.2881

[BIO058612C5] Blumstein, D. T., Mennill, D. J., Clemins, P., Girod, L., Yao, K., Patricelli, G., Deppe, J. L., Krakauer, A. H., Clark, C., Cortopassi, K. A. et al. (2011). Acoustic monitoring in terrestrial environments using microphone arrays: applications, technological considerations and prospectus. *J. Appl. Ecol.* 48, 758-767. 10.1111/j.1365-2664.2011.01993.x

[BIO058612C6] Campos-Cerqueira, M. and Aide, T. M. (2016). Improving distribution data of threatened species by combining acoustic monitoring and occupancy modelling. *Methods Ecol. Evol.* 7, 1340-1348. 10.1111/2041-210X.12599

[BIO058612C7] Cardoso, G. C. and Price, T. D. (2010). Community convergence in bird song. *Evol. Ecol.* 24, 447-461. 10.1007/s10682-009-9317-1

[BIO058612C8] Cavender-Bares, J., Ackerly, D. D., Baum, D. A. and Bazzaz, F. A. (2004). Phylogenetic overdispersion in Floridian oak communities. *Am. Nat.* 163, 823-843. 10.1086/386375 15266381

[BIO058612C9] Chek, A. A., Bogart, J. P. and Lougheed, S. C. (2003). Mating signal partitioning in multi-species assemblages: a null model test using frogs. *Ecol. Lett.* 6, 235-247. 10.1046/j.1461-0248.2003.00420.x

[BIO058612C10] Chitnis, S. S., Rajan, S. and Krishnan, A. (2020). Sympatric wren-warblers partition acoustic signal space and song perch height. *Behav. Ecol.* 31, 559-567. 10.1093/beheco/arz216

[BIO058612C11] Correll, M. D., Strasser, E. H., Green, A. W. and Panjabi, A. O. (2019). Quantifying specialist avifaunal decline in grassland birds of the Northern great plains. *Ecosphere* 10, e02523. 10.1002/ecs2.2523

[BIO058612C12] Dabadghao, P. M. and Shankarnarayan, K. A. (1973). *The Grass Cover of India*. New Delhi: Indian Council of Agricultural Research.

[BIO058612C13] del Hoyo, J., Collar, N. J., Christie, D. A., Elliott, A. and Fishpool, L. D. C. (2014). *HBW and BirdLife International Illustrated Checklist of the Birds of the World*. Lynx Edicions BirdLife International.

[BIO058612C14] Dutta, S., Rahmani, A. R. and Jhala, Y. V. (2011). Running out of time? The great Indian bustard Ardeotis nigriceps–status, viability, and conservation strategies. *Eur. J. Wildl. Res.* 57, 615-625. 10.1007/s10344-010-0472-z

[BIO058612C15] Grimmett, R., Inskipp, C. and Inskipp, T. (1998). *Birds of the Indian Subcontinent*, *1st* edn. A&C Black.

[BIO058612C16] Hallett, L., Avolio, M., Carroll, I., Jones, S., MacDonald, A., Flynn, D., Slaughter, P., Ripplinger, J., Collins, S., Gries, C. et al. (2020). codyn: Community Dynamics Metrics.

[BIO058612C17] Hill, J. M., Egan, J. F., Stauffer, G. E. and Diefenbach, D. R. (2014). Habitat availability is a more plausible explanation than insecticide acute toxicity for U.S. grassland bird species declines. *PLoS ONE* 9, e98064. 10.1371/journal.pone.0098064 24846309 PMC4028314

[BIO058612C18] Ives, A. R. and Helmus, M. R. (2010). Phylogenetic metrics of community similarity. *Am. Nat.* 176, E128-E142. 10.1086/656486 20887187

[BIO058612C19] Jetz, W., Thomas, G. H., Joy, J. B., Hartmann, K. and Mooers, A. O. (2012). The global diversity of birds in space and time. *Nature* 491, 444-448. 10.1038/nature11631 23123857

[BIO058612C20] Jha, R. R. S., Thakuri, J. J., Rahmani, A. R., Dhakal, M., Khongsai, N., Pradhan, N. M. B., Shinde, N., Chauhan, B. K., Talegaonkar, R. K., Barber, I. P. et al. (2018). Distribution, movements, and survival of the critically endangered Bengal Florican Houbaropsis bengalensis in India and Nepal. *J. Ornithol.* 159, 851-866. 10.1007/s10336-018-1552-1

[BIO058612C21] Kaur, M., Joshi, P., Sarma, K. and Das, S. K. (2020). Assessment of plant community structure in Tal Chhapar Wildlife Sanctuary, Rajasthan, India. *Species* 21, 126-139.

[BIO058612C22] Kembel, S. W., Cowan, P. D., Helmus, M. R., Cornwell, W. K., Morlon, H., Ackerly, D. D., Blomberg, S. P. and Webb, C. O. (2010). Picante: R tools for integrating phylogenies and ecology. *Bioinformatics* 26, 1463-1464. 10.1093/bioinformatics/btq166 20395285

[BIO058612C23] Krishnan, A. (2019). Acoustic community structure and seasonal turnover in tropical South Asian birds. *Behav. Ecol.* 30, 1364-1374. 10.1093/beheco/arz087

[BIO058612C24] Krishnan, A. and Tamma, K. (2016). Divergent morphological and acoustic traits in sympatric communities of Asian barbets. *R. Soc. Open Sci.* 3, 160117. 10.1098/rsos.160117 27853589 PMC5108939

[BIO058612C25] Luther, D. A. (2008). Signaller: receiver coordination and the timing of communication in Amazonian birds. *Biol. Lett.* 4, 651-654. 10.1098/rsbl.2008.0406 18832055 PMC2614168

[BIO058612C26] Luther, D. (2009). The influence of the acoustic community on songs of birds in a neotropical rain forest. *Behav. Ecol.* 20, 864-871. 10.1093/beheco/arp074

[BIO058612C27] Marten, K. and Marler, P. (1977). Sound transmission and its significance for animal vocalization - I. Temperate habitats. *Behav. Ecol. Sociobiol.* 2, 271-290. 10.1007/BF00299740

[BIO058612C28] Nelson, D. A. and Marler, P. (1990). The perception of birdsong and an ecological concept of signal space. In *Comparative Perception: Complex Signals*, Vol. 2 ( W. C. Stebbins and M. A. Berkley), pp. 443-478. New York: Wiley.

[BIO058612C29] Nerlekar, A. N. and Veldman, J. W. (2020). High plant diversity and slow assembly of old-growth grasslands. *Proc. Natl. Acad. Sci. USA* 117, 18550-18556. 10.1073/pnas.1922266117 32675246 PMC7414179

[BIO058612C30] Price, T. D., Hooper, D. M., Buchanan, C. D., Johansson, U. S., Tietze, D. T., Alström, P., Olsson, U., Ghosh-Harihar, M., Ishtiaq, F., Gupta, S. K. et al. (2014). Niche filling slows the diversification of Himalayan songbirds. *Nature* 509, 222-225. 10.1038/nature13272 24776798

[BIO058612C31] R Core Team. (2013). R: A language and environment for statistical computing. Mumbai: Bombay Natural History Society.

[BIO058612C32] Rahmani, A. R. (2012). *Threatened Birds of India: Their Conservation Requirements (Bombay Natural History Society)*. New Delhi: Oxford University Press.

[BIO058612C33] Rahmani, A. R. (2016a). D'Ering Memorial Wildlife Sanctuary: Report of Summer surveys in 2016. Mumbai: Bombay Natural History Society.

[BIO058612C34] Rahmani, A. R. (2016b). *Conservation of Threatened Grassland birds of the Brahmaputra floodplains*. Mumbai: Bombay Natural History Society.

[BIO058612C35] Rasmussen, P. C. and Anderton, J. C. (2005). *Birds of South Asia: The Ripley Guide*. Barcelona: Lynx Edicions.

[BIO058612C36] Ratnam, J., Tomlinson, K. W., Rasquinha, D. N. and Sankaran, M. (2016). Savannahs of Asia: antiquity, biogeography, and an uncertain future. *Philos. Trans. R. Soc. B Biol. Sci.* 371, 20150305. 10.1098/rstb.2015.0305 PMC4978864 27502371

[BIO058612C37] Raynor, E. J., Whalen, C. E., Brown, M. B. and Powell, L. A. (2017). Grassland bird community and acoustic complexity appear unaffected by proximity to a wind energy facility in the Nebraska Sandhills. *Condor* 119, 484-496. 10.1650/CONDOR-16-164.1

[BIO058612C38] Ripley, S. D. and Beehler, B. M. (1990). Patterns of Speciation in Indian Birds. *J. Biogeogr.* 17, 639. 10.2307/2845145

[BIO058612C39] Robert, A., Lengagne, T., Melo, M., Gardette, V., Julien, S., Covas, R., Gomez, D. and Doutrelant, C. (2019). The theory of island biogeography and soundscapes: Species diversity and the organization of acoustic communities. *J. Biogeogr.* 46, 1901-1911. 10.1111/jbi.13611

[BIO058612C40] Schmidt, A. K. D., Römer, H. and Riede, K. (2013). Spectral niche segregation and community organization in a tropical cricket assemblage. *Behav. Ecol.* 24, 470-480. 10.1093/beheco/ars187

[BIO058612C41] Smith, B. and Wilson, J. B. (1996). A consumer's guide to evenness indices. *Oikos* 76, 70. 10.2307/3545749

[BIO058612C42] Srinivasan, U., Tamma, K. and Ramakrishnan, U. (2014). Past climate and species ecology drive nested species richness patterns along an east-west axis in the Himalaya. *Glob. Ecol. Biogeogr.* 23, 52-60. 10.1111/geb.12082

[BIO058612C43] Sugai, L. S. M., Silva, T. S. F., Ribeiro, J. W. and Llusia, D. Jr. (2019). Terrestrial passive acoustic monitoring: review and perspectives. *Bioscience* 69, 15-25. 10.1093/biosci/biy140

[BIO058612C44] Tobias, J. A., Planque, R., Cram, D. L. and Seddon, N. (2014). Species interactions and the structure of complex communication networks. *Proc. Natl. Acad. Sci.* 111, 1020-1025. 10.1093/biosci/biy147 24395769 PMC3903186

[BIO058612C45] Tucker, C. M., Cadotte, M. W., Carvalho, S. B., Jonathan Davies, T., Ferrier, S., Fritz, S. A., Grenyer, R., Helmus, M. R., Jin, L. S., Mooers, A. O. et al. (2017). A guide to phylogenetic metrics for conservation, community ecology and macroecology. *Biol. Rev.* 92, 698-715. 10.1111/brv.12252 26785932 PMC5096690

[BIO058612C46] Vickery, P. D. and Herkert, J. R. (1999). *Ecology and Conservation of Grassland birds of the Western Hemisphere* ( P. D. Vickery and J. R. Herkert), Camarillo, CA, USA: Cooper Ornithological Society.

[BIO058612C47] Webb, C. O. (2000). Exploring the phylogenetic structure of ecological communities: an example for rain forest trees. *Am. Nat.* 156, 145-155. 10.1086/303378 10856198

[BIO058612C48] Webb, C. O., Ackerly, D. D., McPeek, M. A. and Donoghue, M. J. (2002). Phylogenies and community ecology. *Annu. Rev. Ecol. Syst.* 33, 475-505. 10.1146/annurev.ecolsys.33.010802.150448

