# Supplementary material for: Convergent acoustic community structure in South Asian dry and wet grassland birds
Source: Biol Open. 2021 Jun 22;10(6):bio058612. doi: 10.1242/bio.058612 (PMC8272033; doi:10.1242/bio.058612)
Supplement: Supplementary information [file biolopen-10-058612-s1.pdf]

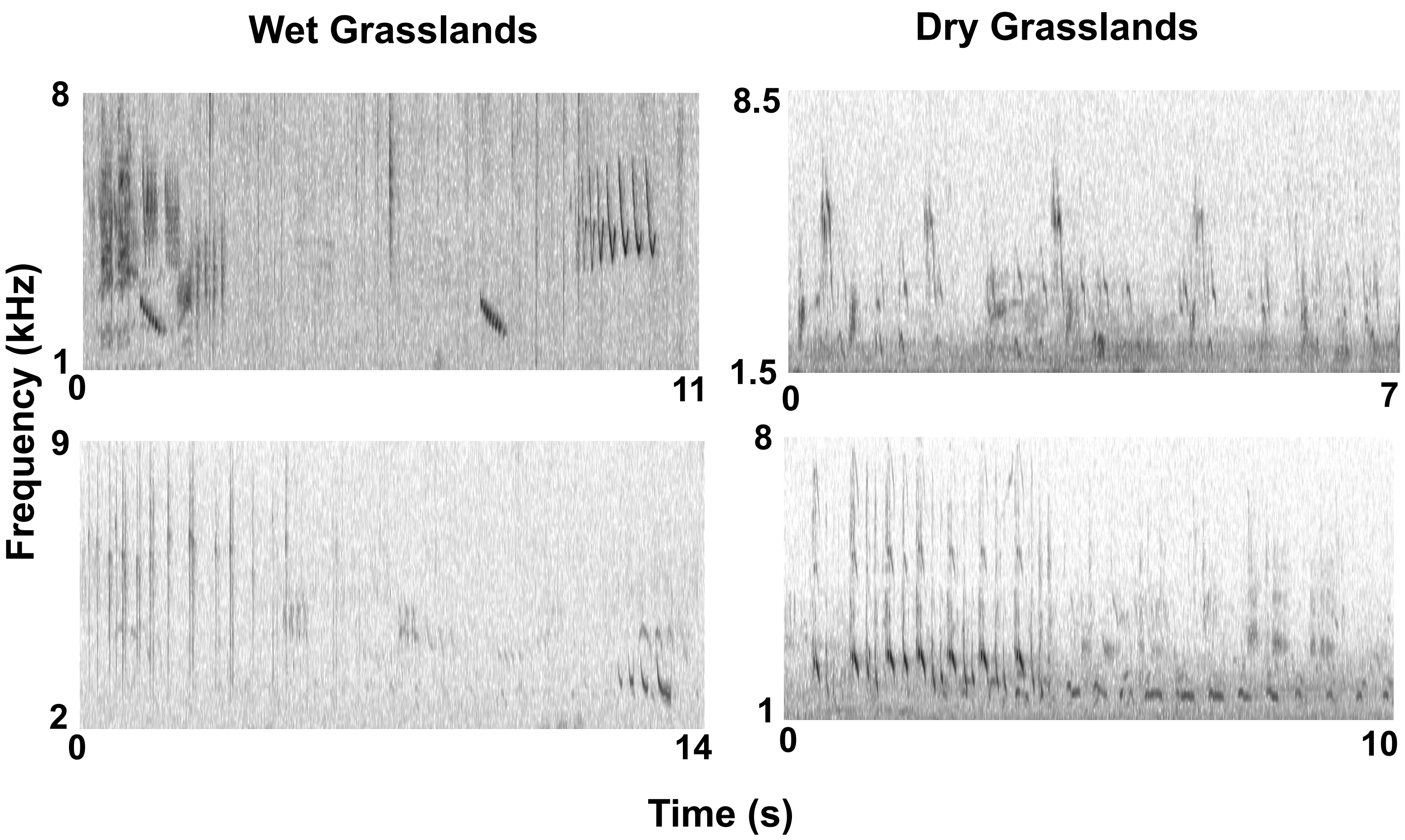

Figure S1. Example spectrograms of passively recorded acoustic data in both habitats, demonstrating the typical range of bird vocal activity we observed.

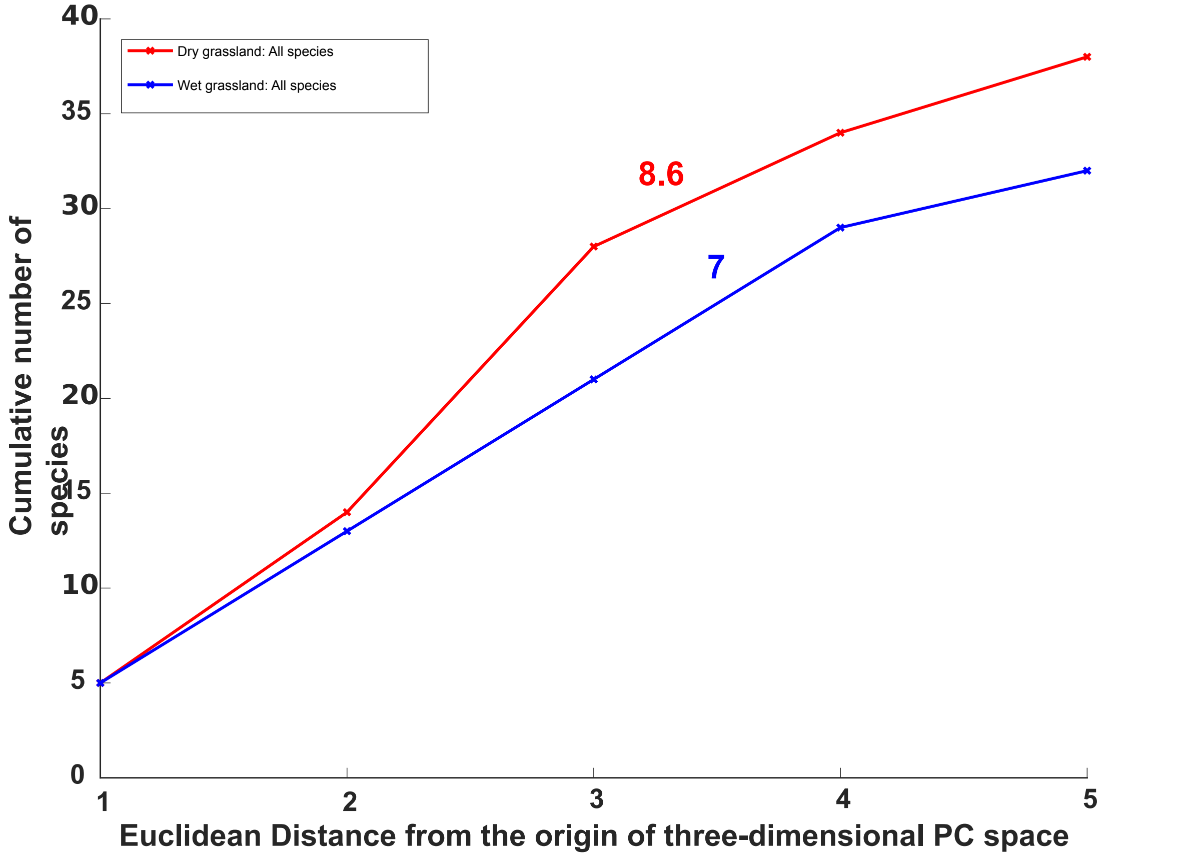

Figure S2: Accumulation of species with distance from the centroid of signal space. The numbers indicate the slopes obtained from a linear fit. Both communities exhibit similar distributions of species, with the main difference coming from the slightly higher number of species in dry grasslands.

Table S1

Click here to download Table S1

Biology Open • Supplementary information
